# Coronavirus Disease 2019 and Primary Antibody Deficiencies. Comment on Milota et al. Clinical Outcome of Coronavirus Disease 2019 in Patients with Primary Antibody Deficiencies. *Pathogens* 2023, *12*, 109

**DOI:** 10.3390/pathogens12040532

**Published:** 2023-03-29

**Authors:** Öner Özdemir

**Affiliations:** Division of Allergy and Immunology, Department of Pediatrics, Research and Training Hospital, Medical Faculty, Sakarya University, Adapazarı, Sakarya 54100, Türkiye; ozdemir_oner@hotmail.com; Tel.: +90-(264)-444-54-00; Fax: +90-(264)-275-91-92

We have read the article titled ‘Clinical Outcome of Coronavirus Disease 2019 in Patients with Primary Antibody Deficiencies’ by Milota et al. with great interest [1]. I have a couple of concerns about the perplexing conclusions from their review of the literature.

First, the interpretation of the literature by the authors is confusing for the readers. In the abstract, the authors say that there are poorer outcomes in primary antibody deficiencies (PADs) compared to other inborn errors of immunities [1]. However, many studies (Israelian, Italian, European, etc.), as mentioned in the manuscript, demonstrated equivocal results, probably affected by demographic differences in their study population and infectiousness and severity of SARS-CoV-2 variants [1]. Furthermore, during their review of the literature, the authors mention that mild to moderate COVID-19 disease development was reported in most PAD patients and the overall infection fatality rate was not much higher than in the general population [1]. Therefore, I think that in the abstract it is better to mention the main risk factors affecting poorer prognosis in PAD patients including older age, comorbidities, PAD-associated complications, and lymphopenia or hypogammaglobulinemia. As a result, the authors should emphasize that poorer outcomes were associated with comorbidities rather than the type of PADs.

Second, the authors cited Milota et al.’s study reporting the largest proportion of infections occurred in patients with CVID, hereditary angioedema (HAE), and unclassified PADs [2]. However, it is necessary to delineate further the situation of HAE disease in the COVID-19 pandemic. Patients with HAE have been thought to be at increased risk for COVID-19 development due to intrinsic dysregulation of the plasma kallikrein-kinin system. However, reports in the literature differ. In a study, the subjects with HAE with C1 inhibitor deficiency (HAE-C1-INH) who were not taking medications (e.g., C1-INH and icatibant) had a significantly higher rate of reported COVID-19. The incidence of reported COVID-19 was not significantly different between the normal controls and the subjects with HAE-C1-INH but was greater in the subjects with HAE with normal C1-INH [3]. COVID-19 severity and complications were similar in all the groups. In this study, comorbidities, e.g., obesity, autoimmune disease, having HAE with normal C1-INH type, and not being on any medication were major risk factors for contracting SARS-CoV-2 infection [3]. Another study showed that there was no significant difference in the frequency, and severity of angioedema attacks during the course of COVID-19 in HAE patients [4]. Consequently, the authors should have discussed the type, comorbidities of HAE, and medication use before stating the largest proportion of SARS-CoV-2 infections that happened in HAE patients.

Third, the authors also mentioned a meta-analysis by Sunjaya et al. concluding that the risk of severe COVID-19 in patients with bronchial asthma was lower compared to non-asthmatic ones [5]. This also seems to be a general conclusion and misleads the readers. Although inhaled corticosteroids and biologics are generally safe and may be associated with a protective effect against severe SARS-CoV-2 infection [6], more severe asthma was related to more severe COVID-19 prognosis, but type 2 inflammation in asthma patients was not [7]. Accordingly, a physician should evaluate the type and severity of asthma as well as the control level of the patient’s asthma before making a decision on the prognosis.
